# *Bartonella* spp. in households with cats: Risk factors for infection in cats and human exposure

**DOI:** 10.1016/j.onehlt.2023.100545

**Published:** 2023-04-19

**Authors:** Paulina Sepúlveda-García, Amir Alabi, Karla Álvarez, Lisbeth Rojas, Armin Mella, Luiz Ricardo Gonçalves, Marcos Rogerio André, Rosangela Zacarias Machado, Ananda Müller, Gustavo Monti

**Affiliations:** a Escuela de Graduados, Facultad de Ciencias Veterinarias, Universidad Austral de Chile, Valdivia, Chile; b Instituto de Medicina Preventiva Veterinaria, Universidad Austral de Chile, Valdivia, Chile; c Department of Pathology, Reproduction and One Health, São Paulo State University (UNESP), Jaboticabal, Brazil; d Instituto de Ciencias Clínicas Veterinaria, Universidad Austral de Chile, Valdivia 5090000, Chile; e Instituto de Bioquímica y Microbiología, Universidad Austral de Chile, Valdivia 5090000, Chile; f One Health Center for Zoonoses and Tropical Veterinary Medicine, Department of Biomedical Sciences, Ross University School of Veterinary Medicine, Basseterre, Saint Kitts and Nevis; g Quantitative Veterinary Epidemiology, Wageningen University & Research, Wageningen 6702 PB, the Netherlands

**Keywords:** Bartonellosis., Cat-scratch disease, Zoonosis, qPCR, Serology, One health, felids

## Abstract

The aim of this study was to estimate the occurrence of *Bartonella* spp. per household in cats and the risk factors for *Bartonella* spp. positivity in cats and their owners from Valdivia, Chile. A total of 464 cats (distributed within 324 households) and 326 humans (control group [*n* = 112] and cat owner [*n* = 214]) distributed in 262 households were sampled. From the cat owners (n = 214), 128 humans were in households where the cat was also sampled, totaling 84 households with dual sampling. Real-time PCR (qPCR) was used for *Bartonella* spp. detection in blood from cats and humans, and immunofluorescent immunoassay (IFA) anti-*Bartonella henselae* was performed in human serum samples. Out of the total of 324 households, 20.43% presented at least one *Bartonella* positive cat. From the households with dual sampling, 29.7% (25/84) presented at least one qPCR-*Bartonella* spp. positive cat. However, *Bartonella* DNA was not amplified in humans, and in 7.3% (6/82) of the households was found at least one of the cat's owners exposed to *B. henselae*. Cats younger than one year (Odds Ratio (OR) = 5.3), non-neutered (OR 3.46), sampled at home (OR 5.82), and with improper application of tick/flea control products (OR 3.13) showed a higher risk for *Bartonella* spp. presence. Humans with occupational exposure involving animal contact, were more likely to exhibit *B. henselae* seropositivity (OR 7.5). *Bartonella* spp. was present in the cats a moderate number of households, but *Bartonella* DNA was not detected in owners' blood, inferring that there is a low risk of recent human infection in the studied population.

## 1 Introduction

One Health is an approach that recognizes that people's health is closely connected to the health of animals and the environment. Whilst the One Health concept is not new, it has become more critical in recent years because many factors have changed interactions between people, animals, and the environment [1]. During recent decades, the number of domestic cats (*Felis silvestris catus*) within households has increased, particularly as cats are one of the most popular companion animals [2]. The presence of cats in the household is important because cats are recognized as reservoirs for several zoonotic agents, primarily parasites, including *Ancylostoma* spp., *Giardia* spp., *Toxocara canis*, *Toxoplasma gondii* and bacteria of which the most recognized are *Leptospira* species*, Yersinia pestis*, and several vector-borne pathogens: *Bartonella* spp., *Coxiella burnetii*, *Ehrlichia* spp., *Rickettsia felis* [3].

The domestic cat is the major reservoir for three zoonotic *Bartonella* species, namely *B. henselae*, *B. clarridgeiae* and *B. koehlerae* [4,5] and cat fleas (*Ctenocephalides felis*) are the primary vector of feline *Bartonella*. In humans, *Bartonella clarridgeiae* is postulated as a minor agent of cat-scratch disease (CSD)[6], *B. koehlerae* is reported as the causal agent for negative blood culture endocarditis [7] and has been associated with the presence of unspecific symptoms such as joint pain, muscle pain, headache, insomnia and memory loss [8]. Finally, *B. henselae* is the genus's primary zoonotic agent responsible for most cases of CSD and clinical manifestation. Although CSD is a benign self-limiting disease that develops as a fever and lymphadenopathy in humans, lately, *B. henselae* has been found in several unspecific health conditions. These include, but are not limited to, central nervous system syndromes [9,10], fever of unknown origin (FUO) [11,12], hepatosplenic disease [[13], [14], [15]], negative blood culture endocarditis [16], neuroretinitis [17,18], musculoskeletal [[19], [20], [21], [22]] and cutaneous lesions [23]. Because of the diverse and unspecific clinical manifestations [26], poor information due to the lack of readily available diagnostic tests, and scarcity of medical research, especially in South American countries, delays in *Bartonella* spp. diagnosis in humans or even an underdiagnosis are common [24,25].

*Bartonella* spp. infection in cats is described as endemic in several countries around the world, with diverse prevalence, namely 1.0% and 11.9% in Spain [27,28], 1.6% and 90.2% in Brazil [29,30], 2.5% and 5.5% in Thailand [31,32], 3.94% and 8.5% in China [33,34], 4.6% in Japan [35], 8.5% in Greece [36], 17.8% in Argentina [37], 18.0% in Chile [38], 20.8% in Paraguay [39], 22.6% in Denmark [40] and 24% in United States [41].

The evidence of zoonotic *Bartonella* circulating among the cat population worldwide has triggered human studies. As such, a seroprevalence of 8.7% was detected in human populations in Spain [42], 9.7% in China [43], 11.4% in Italy [44], 11.5% in Turkey [45], 10.3% in Chile [46], 15% in Korea [47], 16.1% in Sweden [48], 19.8% in Greece [49], and 24.7% in Spain [50]. In addition, in the United States, *Bartonella* infection's importance in public health is well established, with an annual incidence of CSD of 4.7 per 100,000 persons < 65 years, and approximately 500 patients hospitalized each year [51]. However, although there are very few epidemiological surveys aimed at elucidating the risks associated with *Bartonella* exposure in humans [42,43,45,[47], [48], [49], [50]], those that exist show a trend of higher seroprevalence in older individuals [42,43,46]. *Bartonella* infection in the cat population is better understood than in the human population, and most investigations denoted higher infection rates in strays [28,32,34,41,44], younger cats, and those presenting flea infestation [41,52].

Regarding the risk of transmission between humans and cats, one study in Turkey simultaneously evaluated *Bartonella* spp. antibodies on cat/dog owners (11.5%) and their cats [45]. In Chile, 18.1% of owners with positive cats (71.0%) had antibodies against *B. henselae* [53]. To the best of the authors' knowledge, no studies focused on the household to estimate the rate of *Bartonella* detection in cats and humans, nor evaluated the risk factors related to *Bartonella* DNA positivity/exposure in humans living in households with cats. It is crucial to evaluate the risk of *Bartonella* bacteremia/exposure implied by cat ownership. This study aimed to: (1) estimate the frequency of households with *Bartonella* spp. infected cats; (2) describe the risk factors for *Bartonella* spp. bacteremia in domiciled cats, and (3) identify *Bartonella* spp. bacteremia and *Bartonella henselae* exposure in cat owners from Valdivia, Chile.

## 2 Materials and methods

### 2.1 Biological material and study area

The experimental protocol for human sampling and analysis was approved by the Valdivia Health Service (#081/2019). The cat study was approved by the bioethics committee of Universidad Austral de Chile (#353/2019).

A cross-sectional observational analysis and convenience sampling was used in this study in Valdivia city, Chile (39°48′51.2” S 73°14.753′ W) between August 2019 and July 2021. The unit of study is represented by the home origin of the cats and the humans living in the household. The number of people and cats needed to assess the risk factor was estimated based on the following formula [54].

$$m=\frac{p_{0}c_{0}{\left(Z_{1-\propto /2}+Z_{1-\beta }\sqrt{\frac{p_{1}c_{1}}{p_{0}c_{0}}}\right)}^{2}}{{\left(p_{1}-p_{0}\right)}^{2}}$$

Assuming: Proportion of not exposed (p_0_c_0_) = 10%, proportion of exposed (p_1_c_1_) = 20%, Type I error *Z*_1−∝/2_= 5%, type II error (*Z*_1−*β*_)= 10%, then at least 165 people should be sampled. According to the most recent Chilean census [55], there are typically four people living in each house in the region, so we calculated that there should be at least 50 households.

Cat samples were obtained from two sources:

• Cats admitted to veterinary clinics and cats involved in public neutering initiatives carried out in the province of Valdivia. If the owner of the cat sampled at the clinic has other cats at home, they were also included.
• Cats whose owners donated blood at Valdivia's hospital.

In each instance, the owners were made aware of the relevant aspects of the study and their authorization was obtained via an informed consent. In addition, the clinical record for each cat was completed by a veterinarian. A total of 464 cats were sampled, distributed within 324 households.

Human blood samples were obtained from people living with cats and people without cats. These samples were obtained from two sources:

• Cat owners who consented to participate and that their cats were sampled in the clinics or neutering initiatives described above. If additional members of the household agreed to take part, they were also included.
• Those who visited Valdivia hospital as blood donors, who willingly gave their consent to participate, and declared whether they owned cats or not (control group).

**Fig. 1:**
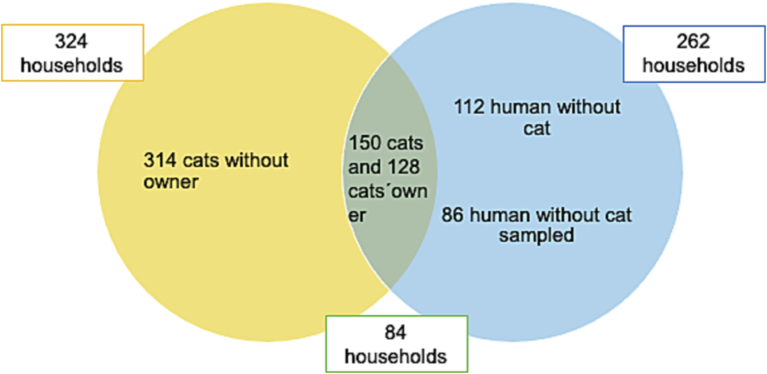
Schematic representation that summarizing the cats and humans sampling procedure.

A total of 326 persons were sampled, distributed as: (1) control group (blood donors living in households without cats (*n* = 112); (2) humans living in households with cats (*n* = 214). A total of 128 people from those living with cats (28 blood donors plus 100 cat owners who visited veterinary clinics) belonged to 84 households, where the cats were also sampled (Fig. 1).

### 2.2 Epidemiological data collection

Two standardized epidemiological questionnaires were delivered with the aim of obtaining information about the cats and the people.

The one for cats contained information like age, gender, reproductive status, cohabitation with another cat, cohabitation with a dog, access outside the house, if the cat suffered injuries (scratch or bite), contact with rodents, and adequate external antiparasitic application.

The second questionnaire, regarding humans, contained information like age, gender, origin, occupational animal exposure, contact with cats, history of cat scratches or bites, presence of suspicious signs of CSD, and history of underlying disease.

In addition, for humans who lived with cats, the following explanatory variables were accessed: the presence of more than one cat in the household, sleeping with the cat, playing with the cats using hands, cats having contact with rodents, reproductive status of the cat, adequate control and treatment of flea/ticks on the cat, presence of one or more cat in household with external access, and living in a household with a *Bartonella* spp. qPCR positive cat.

### 2.3 Blood collection

Cat owners signed a “cat sampling and use of samples” consent form. In addition, sampled humans signed a “human sampling and use of samples” consent form.

Blood samples were aseptically collected from cats as well as humans in EDTA vacutainer tubes. In addition, in humans, a second sample (without anticoagulant), was collected to obtain serum. The collection of human samples was performed by a licensed health professional. In cats, the samples were aseptically collected by a veterinarian via venipuncture of the cephalic vein. Following blood collection, all samples were transported in a cold chain at 4 °C. Whole EDTA blood samples were stored at − 20 °C until PCR analysis, and blood samples without anticoagulant were centrifuged at 1600*g* for ten minutes to separate the serum and store it at − 20 °C, until serological analysis.

### 2.4 DNA extraction/purification

Frozen EDTA blood samples from humans (*n* = 326) and cats (*n* = 464) were thawed at room temperature and vortexed at the UACh Veterinary Clinical Pathology Laboratory, Valdivia, Chile. DNA extraction and purification from 200 μL of blood were performed using E.Z.N.A. Tissue DNA Kit (E.Z.N.A. Omega BioTek®, Norcross, GA, USA), according to the manufacturer's instructions, to obtain 100 μL of purified DNA. DNA concentration and purity were determined (NanoDrop ND-1000 Thermo Scientific©, Waltham, MA, USA). The 260/280 nm absorbance ratio (OD260/OD280) provided an estimate of sample purity. DNA was stored at −20 °C prior to performing PCR assays.

### 2.5 Endogenous control conventional (c) PCR

The encoding interphotoreceptor retinoid-binding protein (IRBP) gene (227 bp) was used as an internal control for a PCR assay for feline and human genomic DNA using primers IRBPF (5′- TCCAACACCACCACTGAGATCTGGAC -3′) and IRBPR (5′- GTGAGGAAGAAATCGGACTGGCC -3′) to discard the presence of PCR inhibitors. The reaction mixture was composed of 12.5 μL Gotaq® Green Master Mix (Promega®, Madison, WI, USA), 500 nM of each primer, and 5 μL of DNA template, bringing the total volume to 25 μL with nuclease-free water (Promega®, Madison, WI, USA). The thermic protocol was: 95 °C for 4 min. Followed by 35 cycles of 94 °C for 30s, 57 °C for 30s, 72 °C for 1 min. and a final extension of 72 °C for 5 min. [56]. As a negative control, all cPCR runs were performed with nuclease-free water (Promega®, Madison, WI, USA). All reactions were performed in a T100™ Thermal Cycler (Bio-Rad, Hercules, CA, USA).

### 2.6 Quantitative real-time (qPCR) for Bartonella spp.

Cat and human DNA samples that resulted in a positive to the IRBP endogenous internal control were submitted to a previously described real-time TaqMan qPCR for *Bartonella* spp. targeting the *nuoG* gene [57]. Amplification reactions were performed in duplicate using 10 μL of PCR mixtures containing 5 μL of Go Taq® Probe qPCR Master Mix, dTTP (Promega, Madison, WI, USA), 1.2 μM of each primer (F-Bart [5’-CAATCTTCTTTTGCTTCACC-3′] and R-Bart [5′- TCAGGGCTTTATGTGAATAC-3′], hydrolysis probe (TexasRed-5’-TTYGTCATTTGAACACG-3’[BHQ2a-Q]3′) and 1 μL of the DNA sample. PCR amplifications were conducted in Low-Profile Multiplate™ Unskirted PCR Plates (BioRad©, Hercules, CA, USA) using a CFX96 Thermal Cycler (BioRad©, Hercules, CA, USA). The amplification conditions were 95 °C for 3 min., followed by 40 cycles of 95 °C for 10 min. and 52.8 °C for 30s. The qPCR was performed following the Minimum Information for Publication of Quantitative Real-Time PCR Experiments (MIQE) [58]. Amplification efficiency (E) was calculated from the slope of the standard curve in each run using the following formula (E = 10^–1/slope^). Copy numbers were estimated using 10-fold serial dilutions of gBlock® (Integrated DNA Technologies, Coralville, IA, USA) encoding the *nuoG B. henselae* sequence (insert containing 83 bp). The number of gBlock® copies was determined according to the formula (Xg/μL DNA/ [gBlock length in bp x 660]) x 6.022 × 10^23^ x gBlock copies/μL. *Bartonella henselae* DNA obtained from a naturally infected cat [59] was used as a positive control. As a negative control, all PCR runs were performed with nuclease-free water (Promega®, Madison, WI, USA). Replicates showing a Cq difference higher than 0.5 were retested.

### 2.7 Serology

Antibodies against *B. henselae* isolates were detected in human serum samples (*n* = 326) using an indirect immunofluorescent-antibody (IFA) assay, according to the procedure described [60] in the IMUNODOT® diagnostic Laboratory, Jaboticabal, Brazil. DH82 cells were inoculated with *B. henselae* isolated from a cat from Brazil (ST9) [61]. The use of DH82 cells infected with *Bartonella* prevents the formation of “clumps” (agglomerations of bacteria) on the IFA slide and increases the homogeneity of the generated antigen.

The flask was incubated for 4–5 days at 37 °C with 5% CO_2_ and, after incubation, the tissue culture was washed twice with calcium and magnesium-free 10× Hank's balanced salt solution (GIBCO-BRL, Gaithersburg, MD) and trypsinized (GIBCO-BRL) for 10 min at 37 °C. The suspended tissue culture was centrifuged at 3000 rpm for 10 min. The supernatant was discarded, and the cells were resuspended in PBS. Posteriorly, the cellular preparations were diluted to achieve a single layer of evenly spaced cells. Formaldehyde (2%) was used for bacteria inactivation and to keep the cellular membranes intact. One hundred and twenty microliters of the cell cultures were spotted onto glass slides (12-well – 10 μL per well), air dried, and then stored at −20 °C until use.

Ten microliters of human serum samples (previously diluted 1:64 in PBS with 5% powdered milk) were added to individual wells of the slide containing the *B. henselae* antigen. Slides were incubated at 37 °C for 30 min., followed by three washes in PBS. Fluorescein-conjugated goat anti-human immunoglobulin G (IgG; ICN Biomedical Inc., Irvine, CA) was diluted in PBS (1:64) with 5% powdered milk containing 5% Evans blue, and 10 μL of the dilution was applied to each well. The slides were incubated at 37 °C for 30 min. and washed three times with PBS + 0.01% Tween 20. Finally, the slides were mounted with coverslips, with the addition of buffered glycerin, and analyzed using a UV microscope at 40× objective (Olympus, BX-FLA). Serum samples from cats were used as negative and positive controls.

### 2.8 Statistical data analysis

The presence of DNA in cat's blood was calculated per household and a household was considered as positive if at least one *Bartonella* spp. qPCR positive cat was found. The apparent prevalence was expressed in percentages with their 95% confidence interval.

For assessing the risk factor associated with *Bartonella* spp. DNA positivity in cats, a generalized mixed-model with binomial errors using households as a random effect was performed. However, for assessing the risk factor associated with *B. henselae* exposure in humans, an ordinary logistic regression was performed.

For both calculations, the general strategy of model constructing was the same. First, all candidate variables were screened through an unconditional analysis with a cut-off of *p* ≤ 0.25. Then, a conditional model was adjusted using the Akaike Information Index (AIC) to assess the model's goodness-of-fit. Interaction and confounding variables were also evaluated. Odds ratios (ORs) and 95% confidence intervals for the ORs were estimated. Unless otherwise stated, *p* ≤ 0.05 was considered statistically significant. The statistical analysis was performed in the software R version 4.1 [62].

## 3 Results

### 3.1 Overall results

A total of 326 human blood samples were obtained, including the control group consisting of 112 blood donors who did not own cats, having a mean age and standard deviation (SD) of 42 ± 17 years old being composed of 52 males (46.4%, [95% CI 37.0–56.0]) and 60 (53.6%, [95% CI 43.9–63.0]) females; and a group of 214 cat owners (114 blood donors living with cats + 100 owners of cats admitted to a veterinary clinic) having a mean age of 33.5 ± 13.6 years old and being composed of 90 males (42.0%, [95% CI 35.4–48.9]) and 124 females (58.0%, [95% CI 51.0–64.1]), with a mean age 33.5 ± 13.6 years old). In addition, 324 households housing a total of 464 domestic cats were sampled. The age group ≥ 1 < 7 years was overrepresented in the cat population (60.4%, [95% CI 55.7–65.0]), and there were about the same numbers of males (47.6%, [95% CI 42.9–52.3]) and females (52.4%, [95% CI 47.7–57.1]), the majority of whom were neutered (63.3%, [95% CI 58.6–68.0]). Eighty-four households were visited for sampling of humans (*n* = 128, [28 blood donors living with cat plus 100 owners of cats admitted to a veterinary clinic]), [mean number of humans per household = 2 and range 1–5]) and their cats (*n* = 150, [mean of cats per household = 2, range 1–9]) living there.

### 3.2 Bartonella qPCR results

All cat DNA samples (*n* = 464) (mean and standard deviation (SD) of DNA concentration = 30.3 ± 3.7 ng/μL; mean and SD 260/280 ratio = 2.0 ± 0.3) and human DNA samples (*n* = 326) (mean and standard deviation (SD) of DNA concentration = 44.0 ± 33.0 ng/μL; mean and SD 260/280 ratio = 1.8 ± 0.3) were positive at PCR targeting IRBP gen.

The *Bartonella*-*nuoG* gene fragment was amplified by qPCR (mean and SD of reactions´ efficiency = 99.9 ± 3.9; r^2^ 0.99 ± 0.004; slope = −3.3 ± 0.09 and Y-intercept = 37.7 ± 1.7) in 79 out of the 464 (17.03% [95% CI, 13.81–20.87]) cat samples. *Bartonella* spp. occurrence by gender was 17.23% (41/238) and 16.74% (36/215) in female and male cats, respectively, the details of *Bartonella* spp. occurrence in cats by group according to the analyzed variables are detailed in Appendix A- Table S1. Out of the 324 households, 66 (20.43% [95% CI, 16.2%–25.3%]) presented at least one *Bartonella* positive cat (1–6 cats infected in a positive house).

Twenty-five of the 84 households (29.8% [95% CI, 20.53–40.87]) where the cat and their owner were sampled, housed at least one *nuoG*-*Bartonella* spp. positive cat. Out of the 150 cats sampled in the 84 households, 30 were *Bartonella* spp. positive (20.0% [95% CI, 16.47–35.85]).

All human samples (*n* = 326) were negative for the amplification of *Bartonella* spp. by qPCR.

### 3.3 *Bartonella henselae* serology results human population

The overall seroprevalence of *B. henselae* in humans was 4.9% [95% CI, 2.92–8.00] (16/326), with titers of 1:64 in all positive samples, and all of them reported past contact with cats. A seroprevalence of 2.68% [95% CI, 0.69–8.21] (3/112) was estimated in the control group (people not owning cats), and 6.07% [95% CI, 3.41–10.40] (13/214) for cat owners. The details of *B. henselae* seroprevalence of the analyzed variables by group are included in Appendix A- Table S2.

Regarding the paired sampling of the cat owner and cat in the same household; in two households the serum sample of the cat owner was not available, then, analysis was performed based on 82 households resulting in six (7.3% [95% CI, 3.0–15.83]) households with at least one cat owner exposed to *B. henselae* and an owner *B. henselae* seroprevalence of 4.68% [95% CI, 1.92–10.35] (6/128). The presence of a *Bartonella* spp. qPCR positive cat along with a seropositive *B. henselae* owner was observed in only one household (Appendix A- Table S3).

### 3.4 Risk factors associated with *Bartonella henselae* exposure in humans

The information derived from surveys of all 326 humans was analyzed to assess risk factors associated with *Bartonella* spp. exposure. Based on the univariate analysis, the variables “live with cats” (*p* = 0.19), “history of cat bite during the two last months” (*p* = 0.21) and “working with animals” (*p* = 0.003) were candidates for a conditional model (Appendix A- Table S4). However, the final model only included the variable “work involving animal contact” as a risk factor for *B. henselae* exposure in humans (OR = 7.5 [95% CI, 1.47–31.62]; *p* = 0.008) (Table 1).

**Table 1:** Multivariate analysis (regression logistic model) of the variables associated with *B. henselae* exposure in humans from Valdivia (*n* = 326).

Variable	Category	Coeff.	S.E.	OR	95% CI	*p*-value
Intercept		−3.59	0.59			<0.01
Live with cats	No	Reference				
	Yes	0.47	0.69	1.61	0.45–7.45	0.49
Laboral risk	No	Reference				
	Yes	2.02	0.76	7.54	1.47–31.62	0.008

### 3.5 Cat ownership conditions as risk factors to *B. henselae* exposure in cat owners

An ordinary logistic regression model analyzed variables concerning cat ownership by households to estimate the risk factor associated with the presence of Bartonella-exposed humans (*n* = 128) in the household. None of the analyzed ownership variables (sleep with the cats, use hands to play with the cats, lives in household with more than one cat, reproductive status of the cat, one or more cat of the household have contact with rodents, adequate control and treatment for flea/tick on the cat, the lifestyle of the cat and living in a household with at least one Bartonella spp. positive cat) were associated with the presence of anti-*B. henselae* antibodies in humans from those households (Table 2).

**Table 2:** Univariate analysis result of risk factor associated with Bartonella exposure in cat owners (n = 128) from the 82 households where both humans and cats were sampled in Valdivia.

	Category	Coeff.	S.E.	OR	95% CI	p-value
Sleep with the cat	No	Reference				
	Yes	−0.52	0.94	0.56	0.09–3.73	0.58
Use the hand like toys for the cat	No	Reference				
	Yes	0.35	1.17	1.42	0.14–14.16	0.76
Live with more than one cat	No	Reference				
	Yes	−0.47	0.93	0.62	0.10–3.86	0.61
All cat neutered	No	Reference				
	Yes	0.32	0.89	1.37	0.24–7.79	0.72
Cat has contact with rodent	No	Reference				
	Yes	−33.50	12,700,000.00	0.00	0 - inf	1.00
Flea control of the cat	No	Reference				
	Yes	0.79	0.84	2.21	0.43–11.46	0.35
Cats with external access	No	Reference				
	Yes	1.77	1.51	5.87	0.30–113.47	0.24
Inhabits with PCR positive cats	No	Reference				
	Yes	−0.66	1.11	0.52	0.06–4.59	0.55

### 3.6 *Risk factor associated with detection of Bartonella* spp. *in domestic cats*

The univariate analysis for *Bartonella* spp. DNA positivity in cats (*n* = 464) denoted that the variables age, reproductive status, the number of cats per house, the lifestyle of the cats, presence of suggestive scratch or bite injuries over the last two months, the location of sampling (veterinary clinics or household) were primarily associated with positive results and later used in the multivariate analysis (Appendix A- Table S5). The final model included four variables (Table 3). The variable age was associated with molecular detection of *Bartonella* spp. in cats, where the cats younger than one year old showed a higher risk (OR 5.32 [95% CI, 2.09–13.53]; *p* = 0.0004) than older cats. In addition, non-neutered cats presented a higher risk (OR 3.46 [95% CI, 1.41–8.49]; *p* = 0.007) than neutered cats. Also, the place of sampling showed a statistically significant difference, with those cats sampled at home denoting a higher risk (OR 5.82 [95% CI, 2.19–15.48]; p = 0.0004) than cats admitted to veterinary clinics. Finally, cats with inappropriate administration of tick/flea control products showed a higher risk (OR 3.13 [95% CI, 1.17–8.39]; *p* = 0.02) of *Bartonella* spp. bacteremia.

**Table 3:** Multivariate analysis of risk factor (MLR) associated with *Bartonella* spp. DNA presence in cats (*n* = 464) from households in Valdivia.

Variable	Category	Coeff.	S.E.	OR	95% CI	p-value
Age	>1 ≤ 7 years old	Reference				
	≤1 years old	1.67	0.48	5.32	2.09–13.53	**0.000442**
	> 7 years old	−0.43	0.66	0.65	0.18–2.35	0.508357
Reproductive status	Neutered	Reference				
	Non- neutered	1.24	0.46	3.46	1.41–8.49	**0.006777**
Place of sampling	Clinics	Reference				
	Home	1.762	0.4986	5.82	2.19–15.48	**0.000409**
More than one cat	No	Reference				
	Yes	−0.5357	0.3974	0.58	0.27–1.28	0.177608
Application of external flea/tick control products	No application	Reference				
	Appropriate	0.7974	0.5479	2.22	0.758–6.50	0.145565
	Non appropriate	1.1407	0.5033	3.13	1.17–8.39	**0.023432**

## 4 Discussion

Several authors recognize *Bartonella* species have a worldwide distribution, which varies according to climatic conditions, where major prevalence is estimated in warmer and higher humidity areas that promote vector development [63]. In this study with owned cats, we estimated a *Bartonella* spp. cat prevalence of 17%, which was similar to *Bartonella* reported in domestic cats in Paraguay (20.8%) [39], Chile (18%) [38], Argentina (17.5%) [37] and Brazil (17%) [64]. Otherwise, lower *Bartonella* prevalence is reported in cats from China (3.94% [33] to 8.5% [34]), Greece (8.5%) [36], Japan (4.6%) [35] and Thailand (2.53%) [32], while higher prevalence is reported in cats from Italy (83.5%) [63]. The discrepancy in *Bartonella* prevalence reported in severall studies could be explained mainly by the climatic variation mentioned above and characteristics of the cat population studied (stray cats, owned cats, shelter cats), based on reports of higher *Bartonella* spp. prevalence in stray cats than in owned ones [32,34].

An overall seroprevalence (cat owners + humans without cats) of 4.9% in humans was found, which was close to the seroprevalence reported in blood donors from Brazil (3.2%) [65] and Sweden (3.0%) [48]. Our estimation of the seroprevalence in cat owners (6.07%) is lower than that described for pet owners from Turkey (11.5%) [45], in veterinary personnel from Spain (37%) [66], in blood donors and patient with musculoskeletal complaints from Poland (23%) [20], in children under 15 years old from Jordan (11%) [67], in children from Italy (25.1%) and in healthy blood donors (11%) from Italy [44]. Those differences in prevalence could be attributed partially to the study population used in the different reports, where lesser prevalence is usually found in healthy people as blood donors [48,65], and higher prevalence is reported in studies that involve occupational risk personnel and children [44,66,67]. In addition, *B. henselae* IFA can show variation in sensitivity according to the procedure of the antigen preparation, as well as the kind of cell culture [68,69] and *B. henselae* strain employed [70], thus the lack of a standardized *B. henselae* IFA technique used in the different studies can limit comparisons. One relevant finding of the present study is that *B. henselae* IFA was based on a *B. henselae* Brazilian strain (ST9) used as the antigen. Since we do not know the antigenicity-difference with Chilean strains, there is a chance that certain local *B. henselae* strains from Chile could not be detected, as it was previously recommended the need to involve a country specific *B. henselae* strain in the development of IFA to enable an accurate diagnosis [70]. Furthermore, another limitation is attributed to the inclusion of only *B. henselae* antigen to assess the exposure in humans. There is previously described cross reactivity of *B. henselae* antibodies with other *Bartonella* species, mainly *B. quintana,* which could yield false positive results [60,71]; therefore, the results should be interpreted with caution.

None of the human DNA samples showed amplification on *nuoG*-*Bartonella* spp. qPCR, indicating an absence of *Bartonella* spp. infection within the studied human population. Nonetheless, this outcome should be carefully interpreted, since this negative result can be explained partially by the lower sensitivity of qPCR technique for detecting *Bartonella* spp. from incidental hosts (humans in the present study) compared with the natural reservoirs (cats in the present study). False negative results can occur due to the low bacterial blood charge in incidental hosts, below the detection limit of the technique [72]. One alternative to improve the sensitivity of the technique and consequently increase the likely detection of *Bartonella* spp. in humans, is incubating blood human samples in a liquid pre-enrichment medium (*Bartonella* alphaproteobacterium growth medium, BAPGM) to promote the replication of *Bartonella* spp., and subsequently the initial bacterial charges, which increases the chance of detection via PCR [[72], [73], [74]].

The integration of negative qPCR results with low IgG anti *B. henselae* titers (1:64) in the serum samples of the analyzed people from the present study could suggest that this population was not actively infected with *B. henselae.* However, another study [69] reported that low IgG antibody titer (between 1:64 and 1:256) are found not only in CSD patients but also in healthy controls. Therefore, low antibody titers alone might suggest the onset or the end of CSD, but may also indicate a prior exposure to *B. henselae* [69]. Based on these results, a low risk of recent human infection in the studied population can be inferred. Similarly, in [75] mentioned that the transmission of *B. henselae* from cat to humans via a scratch is a rare event, considering the number of pet cats and the frequency of cat scratches and bite inflicted by these pets.

Regarding the risk factor to *Bartonella* spp. DNA presence in cats, no significant association was found with the variables gender, multiple cat households, presence of dogs in the household, and rodent contact. Those results are supported by previous studies that reported no significant differences in *Bartonella* spp. bacteremia prevalence in female and male cats from Japan [35], Thailand [32] and Turkey [76], likewise no difference was found in *Bartonella* spp. prevalence among Italian cats living in single cat households and multi-cat households [63].

Notwithstanding the acknowledgement of the involvement of cat fleas (*Ctenocephalides felis*) as the primary vector of feline *Bartonella*, the present study showed an association between the inappropriate application of external antiparasitic treatment and *Bartonella* spp. DNA presence in cats. Inconsistent treatment with insecticides and acaricides that do not match the flea's emergence pattern (each 2–3 months), allow flea eggs deposited continue to develop. Thus newly emerged fleas will continue to populate the home for at least a couple of months after treatment [77], masking the effect of the treatment. The reduced efficacy of these products in controlling flea infestations leads to a higher likelihood of animal exposure to *Bartonella* vectors, as well as, the exposure of vectors to lower insecticide doses, which can lead to insecticide resistance influencing vector competence, as evidenced in mosquitoes and parasites or virus infection [78]. Nonetheless, studies regarding the impact of insecticide resistance on vector competence are scarce, and there are no studies on fleas [78].

In addition, kittens (<1 year of age) had 5.3 more chance of being infected with *Bartonella* spp. than the older age group. Similar observations are reported in other studies, showing a decrease in infection occurrence in cats associated with an increment in age [40]. Similarly, significantly higher positive rates of *Bartonella* spp. were observed in cats < 10 years old (5.6% to 7.2%) than in cats > 10 years old (1.3% to 1.7%, *P* = 0.019 to 0.025) [34]. Those observations can be justified by a lower immune response in cats under 12 months of age, who did not reach adequate level of immunoglobulins (IgA, IgG and IgM), opposed to an adult cat [79]. This could favor the infection and persistence by *Bartonella* spp., considering evidence of the participation of the humoral immune response in the elimination of the bacteria in cats [80]. Non-neutered cats from our study had a 3.5 more chance of being infected with *Bartonella* spp., and this fact could be explained by their implicit behavior related to fights and sexual behavior, potentially increasing contact with infected animals and the vectors [81]. The cats sampled at home showed a higher risk of *B. henselae* DNA presence in blood than cats sampled at veterinary clinics (OR = 5.8; *p* = 0.0004). The veterinary attention may be a reflection of socioeconomics factor such household's wealth, indirectly impacting feline health [82], however this result should be interpreted with caution, since frequency of veterinary attendance of the cats were not assessed, and probably some cats sampled at home frequently attended veterinary clinics.

The present study identified working with animals as an occupational hazard for *B. henselae* exposure in humans (OR = 7.5; *p* = 0.008). Correspondingly, a significantly higher *Bartonella* spp. risk was observed in veterinarians from Poland (OR = 2.5) [83], and a high seroprevalence was described in subjects with occupational risk in Chile (69.5%) [84]. In this study, even though a higher percentage of *Bartonella* spp. exposure was evidenced in cat owners (6.1%) than in subjects without cats (2.7%); this difference was not statistically significant, and no association was established between cat ownership and *Bartonella* exposure in humans. Similarly, some studies from Greece [49] and Poland [20] showed that individuals who owned cats were not more likely to be *B. henselae* seropositive. Opposed conclusions were reported in Poland, where specific *B. henselae* antibodies were detected more frequently in cat owners than in the control group [83]. Likewise, in Sweden, owned cats showed a significant association (*p* = 0.002) with *Bartonella* exposure when using 1:128 as the serology cut off (OR = 2.42); however, no significant association was found when using 1:64 as the cutoff [48].

In our study, all humans with a *Bartonella* –IFA positive result reported contact with cats. Nonetheless, no significant association was found between the history of contact with cats and *Bartonella* exposure in humans. Conversely, in a study performed in Brazil [65], the subjects with cat contact, or a past tick bite, were approximately 3 to 4 times more likely to have a *Bartonella* spp. infection than subjects without cat contact or lack of history of a tick bite.

The present study reports the detection of antibodies anti-*B. henselae* in serum samples of healthy human population, and the variable “presence of symptoms suggesting of typical and atypical CSD” was not a risk factor for *B. henselae* exposure in humans. Similarly, IFA seroreactivity to *B. henselae* was not statistically associated with any specific symptoms of CSD in a sanitary workers from Spain [85]. The results reflect that exposure to *Bartonella* spp. in humans does not always mean active disease, and that the bacteria can be present in a healthy human population. This is reinforced by a report of exposure to *B. henselae* in healthy blood donors from several countries (23%) as well as in Poland (24%) [20,83]. In addition, another study using blood donors from Sweden, estimated a seroprevalence of 7.5% and 11.3% for *B. henselae* Houston-1 and *B. henselae* Marseille, respectively [48]. In Brazil, *Bartonella* spp. bloodstream infection was detected in 3.2% of healthy blood donors with confirmation of *B. henselae* bacteremia in six healthy donors by bacterial isolation [65] and in 13.6% of healthy blood donors from Chile [86].

From households where cats and their owners were sampled, 7.3% presented at least one cat owner *B. henselae*-exposed and 29.7% presented at least one *Bartonella* spp. positive cat; in addition, no association was observed among inhabitants of households with at least one *Bartonella* spp. positive cat and the presence of *B. henselae* antibodies in the cat owner. Similarly, authors [83] that tested simultaneously blood cultures and serology of cat owners and their pets, did not find correlations among the infectious status of the owner and their cat.

Our results uncover the importance of other sources of infection or mechanisms of *B. henselae* transmission (besides a cat scratch or bite) to people, for example, a direct flea or tick bite. A CSD case associated with a flea bite was described in Japan [87]. In addition, previous studies describe potential *B. henselae* vector competence of tick *Ixodes ricinus* [88] and *Rhipicephalus sanguineous* [89], as evidenced by the transstadial transmission of *B. henselae* in those specimens. *Bartonella henselae* bacteremia was also reported in two symptomatic family members who denied contact with cats or cat fleas but reported a history of tick bites [90]. Although *I. ricinus* is not described in Chile, other Ixodidae species have been identified, mainly in rodents. So far, there are not studies that evidence *B. henselae* presence in these ticks in Chile. Moreover, the potential participation of dogs as reservoir and source of *B. henselae* infection to humans [91,92] was recently described.

## 5 Conclusions

The present study revealed that, despite a moderate prevalence of households with *Bartonella* spp. infected cats, a low seroprevalence was observed in cat owners, and no-correlation among exposure to *B. henselae* in cat owners and infected cats in the households was found. However, working with animals was confirmed as an occupational hazard for *B. henselae* exposure and should be further investigated. Among the humans exposed to *B. henselae,* no clinical signs were observed. Based on this result, it is suggested that there is a low risk of transmission of *Bartonella* through infected cats to their owner and multiple factors are likely linked to the transmission. Otherwise, non-neutered, young cats with improper acaricide treatment and sampled at home were more at risk for detection of *Bartonella* spp. DNA.

## Funding

This research was funded by “Fondo Nacional de Desarrollo Científico y Tecnológico, grant number 1191462” and by “Beca Doctorado Nacional de Agencia Nacional de Investigación y Desarrollo (21180370), Dirección de posgrado Universidad Austral (Beca graduación oportuna)”.

## Institutional review board statement

The study was approved by the “Ethics Committee of Health Service from Valdivia, Chile (protocol code 081 and date of approval December 18, 2019).” for studies involving humans. And the animal study protocol was approved by “Ethics Committee of Austral University, Chile (protocol code 353/2019 and date of approval May 26, 2019).”

## CRediT authorship contribution statement

**Paulina Sepúlveda-García:** Conceptualization, Software, Data curation, Writing – original draft. **Amir Alabi:** Methodology. **Karla Álvarez:** Methodology. **Lisbeth Rojas:** Methodology. **Armin Mella:** Methodology. **Luiz Ricardo Gonçalves:** Formal analysis. **Marcos Rogerio André:** Investigation, Resources, Writing – review & editing. **Rosangela Zacarias Machado:** Investigation, Resources. **Ananda Muller:** Conceptualization, Validation, Resources, Writing – review & editing, Supervision, Project administration, Funding acquisition. **Gustavo Monti:** Conceptualization, Validation, Data curation, Writing – review & editing, Supervision.

## Declaration of Competing Interest

The authors declare that there is no conflict of interest.

## Data Availability

The authors do not have permission to share data.
