# Supplementary material for: Bartonella spp. in households with cats: Risk factors for infection in cats and human exposure
Source: One Health. 2023 Apr 19;16:100545. doi: 10.1016/j.onehlt.2023.100545 (PMC10288095; doi:10.1016/j.onehlt.2023.100545)
Supplement: Supplementary material [file mmc1.docx]

Table S1: Proportion of Bartonella nuoG-qPCR positive cats (n=464) from Valdivia, by groups of variables to be analyzed by logistic regression analysis.

| **% Bartonella Positive cats (n=464)** | | | |
| --- | --- | --- | --- |
|  | **Category** | **% (n positive/n group)** | **IC 95%** |
| Age | ≤1 years old | 38.0 (41/108) | 28.5 – 47.8 |
|  | >1 ≤ 7 years old | 12.0 (32/267) | 8.5 - 16.6 |
|  | > 7 years old | 6.1 (4/66) | 1.9 - 15.5 |
| Gender | female | 17.2 (41/238) | 12.8 - 22.8 |
|  | male | 16.7 (36/215) | 12.1 - 22.6 |
| Reproductive status | non-neutered | 28.3 (47/166) | 21.7 - 36.0 |
|  | neutered | 10.5 (30/285) | 7.3 - 14.8 |
| Cats number in the house | only one | 21.2 (29/137) | 14.8 - 29.1 |
|  | more than one | 14.9 (48/322) | 11.3 - 19.4 |
| Inhabit with dog in household | no | 19.0 (45/237) | 14.3 - 24.7 |
|  | yes | 14.7 (33/225) | 10.4 - 20.1 |
| Rodent contact | no | 17.8 (51/287) | 13.6 - 22.8 |
|  | yes | 14.8 (18/122) | 9.2 - 22.6 |
| Lifestyle | indoor | 20.0 (26/130) | 13.7 - 28.1 |
|  | outdoor | 29.2 (12/41) | 16.6 - 45.7 |
|  | mixed | 13.7 (40/290) | 10.1 - 18.4 |
| Suffered injuries in the las 2 months | no | 17.4 (57/327) | 13.6 - 22.1 |
|  | yes | 14.4 (17/118) | 8.9 - 22.3 |
| Application of tick/flea control product | no application | 11.94 (16/134) | 7.2 - 18.9 |
|  | propper application | 17.52 (24/137) | 11.8 - 25.1 |
|  | inapropper application | 20.21 (39/193) | 14.9 - 26.7 |
| Sampling location | households | 27.50 (41/149) | 20.8 - 35.7 |
|  | veterinary clinics | 12.10 (38/314) | 8.8 - 16.3 |

Table S2: Proportion of Bartonella exposure in human (with and without cats, n= 326) by variables to be analyzed by logistic regression analysis.

| **% Bartonella exposure human (all human (n= 326)** | | | | | | |
| --- | --- | --- | --- | --- | --- | --- |
|  | **Category** | **% (n positive/n group)** | | | **IC 95%** | |
| Gender | female | 5.4 (10/184) | | | 2.8 - 10.0 | |
| male | 4.2 (6/142) | | | 1.7 - 9.4 | |  |
| Age | <18 | 0.0 (0/9) | | | 0.0 - 37.1 | |
| ≥18 | 5.0 (16/317) | | | 3.0 - 8.2 | |  |
| Work risk | no | 3.7 (11/301) | | | 0.02 - 0.07 | |
| yes | 25.0 (3/12) | | | 6.7 - 57.2 | |  |
| Live with cats | no | 2.7 (3/112) | | | 0.7 - 8.2 | |
| yes | 6.1 (13/214) | | | 3.4 - 10.4 | |  |
| Dogs | no | 5.4 (8/148) | | | 2.5 - 10.7 | |
| yes | 4.1 (7/173) | | | 1.8 - 8.4 | |  |
| Contact with cats | no | 0.0 (0/76) | | | 0.0 - 6.0 | |
| yes | 6.4 (16/249) | | | 3.8 - 10.4 | |  |
| Contact with dogs | no | 4.2 (3/72) | | | 1.1 - 12.5 | |
| yes | 4.8 (12/249) | | | 2.6 - 8.5 | |  |
| Scratch by cat | no | 5.1 (8/164) | | | 2.3 - 9.7 | |
| yes | 4.5 (7/155) | | | 2.0 - 9.4 | |  |
| Bite by cats | no | 3.8 (8/213) | | | 1.8 - 7.5 | |
| yes | 7.1 (7/99) | | | 3.1 - 14.5 | |  |
| CSD clasic | No | 51 (15/297) | | | 2.9 -8.3 | |
| Yes | 0 (0/25) | | | 0.0 – 0.16 | |  |
| CSD atypical | No | 4.2 (5/120) | | | 1.5 - 9.9 | |
| yes | 4.9 (10/202) | | | 2.5 – 9.2 | |  |
|  |  | |  | |  |  |

Table S3: Proportion of Bartonella exposure cat owner belong to the households where the cats sampled where obtained to (n= 128). The proportion of Bartonella-IFA.

| **% Bartonella exposure human** | | | |
| --- | --- | --- | --- |
|  | **Category** | **% (n positive/n group)** | **IC95%** |
| Sleep with the cat | No | 5.7 (2/35) | 1.0 -20.5 |
|  | Yes | 3.5 (3/86) | 0.9 - 10.6 |
| Use the hand like toys for the cat | No | 2.6 (1/39) | 0.1 - 15.1 |
|  | Yes | 3.6 (3/83) | 0.9 - 10.9 |
| PCR positive cats | No | 5.3 (5/94) | 2.0 - 12.5 |
|  | Yes | 2.9 (1/34) | 0.2 - 17.1 |
| More than one cat | No | 5.0 (3/60) | 1.3 - 14.8 |
|  | Yes | 3.2 (2/63) | 0.6 - 11.9 |
| All cat neutered | No | 4 (2/50) | 0.7 - 14.8 |
|  | Yes | 5.4 (4/74) | 1.7 - 13.9 |
| Cats with external access | No | 0 (0/26) | 0.0 - 16.0 |
|  | Yes | 5.9 (6/102) | 2.4 -12.9 |
| Flea control of all cats | No | 3.4 (3/87) | 4.3 - 17.8 |
|  | Yes | 7.3 (3/41) | 1.9 - 21.0 |
| Cats have contact with rodent | No | 7.4 (6/81) | 3.0 - 16.0 |
|  | Yes | 0.0 (0/27) | 0.0 - 15.5 |

Table S4: Univariate analysis of the potential variables associated with B. henselae exposure in human from Valdivia (n=326).

| **Risk factor** | **Category** | **Coeff.** | **S.E.** | **OR** | **95% CI** | **p-value** |
| --- | --- | --- | --- | --- | --- | --- |
| Live with cats | No | Reference |  |  |  |  |
|  | Yes | 0.85 | 0.65 | 2.34 | 0.654 - 8.364 | **0.19** |
| Live with dogs | No | Reference |  |  |  |  |
|  | Yes | -0.30 | 0.53 | 0.74 | 0.261 - 2.086 | 0.57 |
| Gender | Female | Reference |  |  |  |  |
|  | Male | -0.26 | 0.53 | 0.77 | 0.272 - 2.165 | 0.62 |
| Contact with cats | No | Reference |  |  |  |  |
|  | Yes | 16.89 | 1233.57 | 21638831.00 | 0 - inf | 0.99 |
| Contact with dogs | No | Reference |  |  |  |  |
|  | Yes | 0.15 | 0.66 | 1.17 | 0.319 - 4.243 | 0.82 |
| Scratch by cats | No | Reference |  |  |  |  |
|  | Yes | -0.08 | 0.53 | 0.92 | 0.326 - 2.607 | 0.88 |
| Bite by cats | No | Reference |  |  |  |  |
|  | Yes | 0.67 | 0.53 | 1.95 | 0.686 - 1.456 | **0.21** |
| C.S.D. classic symptoms | No | Reference |  |  |  |  |
|  | Yes | -15.63 | 1304.53 | 0.00 | 0 - inf | 0.99 |
| C.S.D. atypical symptoms | No | Reference |  |  |  |  |
|  | Yes | 0.18 | 0.56 | 1.20 | 0.399 - 3.592 | 0.75 |
| Age | < 18 years old | Reference |  |  |  |  |
|  | ≥18 years old | 14.63 | 1318.73 | 2258023.00 | 0 - inf | 0.99 |
| Work with animals | No | Reference |  |  |  |  |
|  | Yes | 2.17 | 0.73 | 8.76 | 2.077 - 36.915 | **0.00** |

Table S5: Univariate analysis of the potential variables associated with Bartonella spp. infection in cats of households from Valdivia (n=464).

| **variables** | **Category** | **Coeff** | **S.E.** | **OR** | **95% CI** | **p-value** |
| --- | --- | --- | --- | --- | --- | --- |
| Age | 1- >1 ≤ 7 years old (1) | Reference |  |  |  |  |
| 0- ≤1 years old (0) | 1.85 | 0.43 | 15.72 | 7.77 - 36.52 | 0.00 |  |
| 2- > 7 years old (2) | -0.76 | 0.60 | 46.76 | 14.44 - 151.59 | 0.21 |  |
| Gender | Female | Reference |  |  |  |  |
| Male | 0.62 | 0.56 | 53.79 | 62.03 - 557.11 | 0.27 |  |
| Reproductive status | Neutered | Reference |  |  |  |  |
| Non-neutered | 1.50 | 0.38 | 448.16 | 212.80 - 943.85 | 0.00 |  |
| Cats number in the house | One cat | Reference |  |  |  |  |
| More than one cat | -0.60 | 0.37 | 54.88 | 26.57 - 113.34 | 0.09 |  |
| Inhabit with dog in household | No | Reference |  |  |  |  |
| Yes | -0.29 | 0.88 | 74.83 | 13.33 - 419.88 | 0.74 |  |
| Lifestyle | Indoor | Reference |  |  |  |  |
| Outdoor | 0.20 | 1.33 | 122.14 | 9.01 - 1655.68 | 0.88 |  |
| Mix | -1.85 | 0.86 | 15.88 | 2.94 - 85.69 | 0.03 |  |
| Suffered injuries in the las 2 months | No | Reference |  |  |  |  |
| Yes | -1.16 | 0.88 | 31.66 | 5.64 - 177.67 | 0.18 |  |
| Application of tick/flea control product | Non application | Reference |  |  |  |  |
| Appropriate application | 0.51 | 0.46 | 166.53 | 67.6 - 410.25 | 0.27 |  |
|  | Non appropriate application | 0.65 | 0.42 | 191.55 | 84.10 - 436.32 | 0.12 |
| Sampling location | Household | Reference |  |  |  |  |
| Vet. Clinics | -1.88 | 0.95 | 15.26 | 2.37 - 98.22 | 0.05 |  |
